# Predictors of Recurrence and Overall Survival in Breast Cancer Patients Undergoing Neoadjuvant Chemotherapy and Surgery: A Comprehensive Statistical Analysis

**DOI:** 10.3390/cancers17060924

**Published:** 2025-03-08

**Authors:** Vlad Bogdan Varzaru, Roxana Popescu, Daliborca Cristina Vlad, Cristian Sebastian Vlad, Aurica Elisabeta Moatar, Andreas Rempen, Ionut Marcel Cobec

**Affiliations:** 1 Doctoral School, Faculty of Medicine, “Victor Babes” University of Medicine and Pharmacy Timisoara, 300041 Timisoara, Romania; 2 ANAPATMOL Research Center, Faculty of Medicine, “Victor Babes” University of Medicine and Pharmacy Timisoara, 300041 Timisoara, Romania; 3 Clinic of Obstetrics and Gynecology, Diakoneo Diak Klinikum, 74523 Schwäbisch Hall, Germany; 4 Department of Cell and Molecular Biology, “Victor Babes” University of Medicine and Pharmacy Timisoara, 300041 Timisoara, Romania; 5 Department of Pharmacology, “Victor Babes” University of Medicine and Pharmacy Timisoara, 300041 Timisoara, Romania; 6 Clinic of Internal Medicine-Cardiology, Klinikum Freudenstadt, 72250 Freudenstadt, Germany; 7 Clinic of Obstetrics and Gynecology, Klinikum Freudenstadt, 72250 Freudenstadt, Germany

**Keywords:** breast cancer, molecular subtype, neoadjuvant chemotherapy

## Abstract

**Background/Objectives**: This study evaluates the impact of clinical, pathological, and treatment-related factors on breast cancer recurrence and overall survival following neoadjuvant chemotherapy and surgery. **Patients and Method:** A total of 298 patients treated at Diakoneo Diak Klinikum, Schwäbisch Hall, Germany (2010–2021) were analyzed. Key variables included hormone receptor status, molecular subtypes, tumor grade, treatment protocols, and metastatic disease at diagnosis. **Results:** Recurrence was strongly associated with metastatic disease (*p* < 0.001) but not with hormone receptor status or molecular subtypes. Platinum/taxane-based chemotherapy was linked to a lower recurrence risk (*p* = 0.05) compared to anthracycline-based regimens. Patients with recurrence had significantly lower overall survival (27.91% vs. 8.24%, *p* < 0.001). Logistic regression suggested a trend toward increased recurrence in ER-positive and PR-negative patients, though not statistically significant. These findings emphasize the importance of personalized treatment strategies and highlight the need for future studies incorporating genomic data and residual disease analysis to refine recurrence risk prediction and therapy selection.

## Simple Summary

Breast cancer is one of the most common cancers worldwide, and despite advances in treatment, many patients still experience cancer recurrence, which can significantly impact survival. This study investigates how different factors—such as tumor characteristics, hormone receptor status, and chemotherapy regimens—affect the likelihood of cancer returning after neoadjuvant chemotherapy and surgery. By analyzing data from nearly 300 patients treated in Germany, we aim to identify patterns that could help doctors predict recurrence risk more accurately. Our findings suggest that certain chemotherapy regimens may lower the risk of recurrence and that some tumor types may have a higher chance of returning. Understanding these factors can lead to more personalized treatment approaches, helping doctors choose the best therapies for each patient. This research contributes to improving breast cancer care by refining treatment strategies and reducing the chances of recurrence, ultimately improving patient outcomes.

## 1. Introduction

Breast cancer is the most frequently diagnosed malignancy in women worldwide, with over 2.26 million new cases reported in 2020 [1]. Despite advances in early detection and treatment, breast cancer remains a leading cause of cancer-related mortality, largely due to recurrence and metastasis. Treatment strategies have evolved significantly over the years, incorporating multimodal approaches to improve patient outcomes. Among these, neoadjuvant chemotherapy (NAC) has gained widespread acceptance, particularly in patients with locally advanced and high-risk diseases. NAC not only reduces tumor burden, facilitating breast-conserving surgery (BCS), but also provides insight into tumor biology and chemosensitivity, guiding further treatment decisions [2].

A crucial aspect of modern breast cancer management is the use of advanced imaging techniques for diagnosis, monitoring, and surgical planning. Preoperative imaging plays a key role in assessing tumor response to NAC, influencing the decision between BCS and mastectomy, and improving surgical precision. Mammography and ultrasound are commonly used in routine screening and staging, while magnetic resonance imaging (MRI) is particularly valuable for detecting multifocal disease and residual tumor tissue. Additionally, computed tomography (CT) and positron emission tomography (PET-CT) are essential tools for staging and evaluating distant metastases. Recent studies highlight the role of imaging not only in tumor localization but also in predicting treatment response and recurrence risk, ultimately optimizing surgical outcomes and reducing the likelihood of incomplete resections [3,4].

Despite these advancements, breast cancer recurrence remains a significant challenge, influenced by a complex interplay of tumor biology, treatment response, and patient-specific factors. Molecular subtypes, hormone receptor status, and chemotherapy regimens all contribute to variability in recurrence rates and overall prognosis. However, identifying reliable predictors of recurrence and survival remains an area of active research. Understanding these prognostic markers is essential for developing personalized treatment strategies, particularly for patients at higher risk of recurrence [5,6].

This study aims to evaluate the impact of clinical, pathological, and treatment-related factors on breast cancer recurrence and overall survival in patients undergoing NAC followed by surgery. By analyzing key variables such as hormone receptor status, molecular subtypes, tumor grade, and chemotherapy regimens, we seek to provide insights into recurrence patterns and prognostic factors, ultimately contributing to more effective, individualized treatment strategies for breast cancer patients.

## 2. Materials and Methods

In our study, we conducted a single-center retrospective analysis using anonymized breast cancer data recorded between 1 January 2010 and 31 December 2021, at the Clinic of Obstetrics and Gynecology, Diakoneo Diak Klinikum Schwäbisch Hall, Germany.

Our analysis focused exclusively on female breast cancer patients who underwent neoadjuvant chemotherapy followed by surgery. The inclusion criteria encompassed all newly diagnosed breast cancer patients who received neoadjuvant chemotherapy and had complete clinical data. Conversely, the exclusion criteria applied to patients with incomplete data or those who did not have an indication for neoadjuvant chemotherapy. For most patients requiring neoadjuvant chemotherapy (NAC), treatment consists of an anthracycline-based regimen followed by taxane-based therapy. In triple-negative breast cancer (TNBC) and HER2-positive disease, additional agents such as platinum-based chemotherapy and anti-HER2 therapy are included. Epirubicin is administered at a dose of 90 mg/m^2^ as an intravenous infusion on day 1 of each cycle. Cyclophosphamide is given at a dose of 600 mg/m^2^ intravenously on day 1 of each cycle. This combination is repeated every 3 weeks for a total of 4 cycles, depending on the patient’s tolerance and risk profile. After completion of EC, treatment continues with paclitaxel at a dose of 80 mg/m^2^, given as a weekly infusion for 12 cycles. For patients with triple-negative breast cancer (TNBC), carboplatin is added to the standard regimen to improve response rates, particularly in patients with suspected or confirmed BRCA1/2 mutations. Carboplatin is administered at AUC 5–6 intravenously every 3 weeks for 4 to 6 cycles, in combination with paclitaxel. For patients with HER2-positive breast cancer, neoadjuvant chemotherapy includes dual anti-HER2 therapy with trastuzumab (Herceptin) and pertuzumab, combined with taxane-based chemotherapy. Trastuzumab is given as an initial loading dose of 8 mg/kg, followed by 6 mg/kg every 3 weeks as a maintenance dose. Pertuzumab is administered at an initial dose of 840 mg, followed by 420 mg every 3 weeks. After surgery, HER2-targeted therapy is continued for up to one year as part of adjuvant treatment.

The analysis aimed to identify significant differences between recurrence and no-recurrence groups, assess survival probabilities across key clinical and pathological variables, and determine predictors of overall survival using multivariate models. We began by summarizing continuous variables using medians and interquartile ranges (IQRs) and categorical variables using counts and percentages. The normality of continuous variables was assessed using the Shapiro–Wilk test, which guided the selection of appropriate statistical methods. Differences between recurrence and no-recurrence groups were evaluated using the Pearson Chi-Squared test for categorical variables and the Mann–Whitney U test for continuous variables, ensuring robust comparisons in the presence of non-normal data. To explore factors associated with recurrence, a logistic regression model was constructed, incorporating key predictors such as diagnostic groups, treatment protocols, hormone receptor status, HER-2/neu positivity, and age. Odds ratios (ORs) with 95% confidence intervals (CIs) were reported to assess the strength and direction of associations. Interaction terms were also included to investigate combined effects between variables, such as HER-2/neu status and treatment protocols, as well as age at diagnosis and PR status, on recurrence risk.

Survival outcomes were further analyzed using Kaplan–Meier survival curves to compare survival probabilities between groups, with death as the event and time to event measured from diagnosis. Differences in survival probabilities were evaluated using the log-rank test. Significant variables identified in the Kaplan–Meier analysis were further examined in a Cox Proportional Hazards (Cox-PH) model, allowing for multivariate analysis of predictors of overall survival. Hazard ratios (HR) with 95% CIs were reported to quantify the impact of recurrence and HER-2/neu status on survival outcomes.

To ensure interpretability and clarity, visualizations such as Kaplan–Meier plots, interaction plots, and bar charts were used to illustrate key findings, highlight differences in recurrence rates, and depict survival trajectories across subgroups. All statistical analyses were performed at a significance threshold of *p* < 0.05 using R (version 4.3.0) and RStudio (version 2023.06.0+421), ensuring the reproducibility and robustness of the results.

A post hoc power analysis was conducted to assess the statistical power of our study given the observed sample sizes. The total study cohort consisted of 298 patients, with subgroup distributions as follows: anthracycline-based chemotherapy (n = 258), platinum/taxane-based chemotherapy (n = 30), recurrence group (n = 43), and no-recurrence group (n = 255). The analysis indicated that, given the subgroup sizes, the power to detect small-to-moderate effect sizes was below the conventional threshold of 80% in several comparisons, particularly for the platinum/taxane-based chemotherapy group and the recurrence group. While meaningful trends were observed, the study may have been underpowered to detect certain statistically significant differences. The results should be interpreted with this limitation in mind.

To account for potential confounding factors, the analysis included adjustments for key tumor characteristics, such as tumor grade and stage, which are known prognostic factors influencing recurrence and survival. However, data on surgical approach (breast-conserving surgery vs. mastectomy) and adjuvant therapies (radiotherapy, endocrine therapy) were not available in our dataset and were therefore not included in the analysis. While these factors could influence treatment outcomes, our study primarily focused on the impact of chemotherapy regimens on recurrence and survival.

## 3. Results

The study cohort consisted of 298 patients diagnosed with breast cancer who underwent neoadjuvant chemotherapy followed by surgery. The primary focus of the study was to evaluate recurrence rates and overall survival.

The variables analyzed included both categorical and continuous clinical and pathological factors. Categorical variables included the affected breast (left or right), hormone receptor status (ER, PR, and HER2/neu), molecular subtypes (e.g., Luminal A, Luminal B, Basal-like), tumor grade (G1–G3), diagnostic categories (e.g., metastatic disease, primary tumor without metastasis, triple-negative status), and treatment protocols (anthracycline-based or platinum/taxane-based regimens). Mortality data were also recorded.

Continuous variables included age at diagnosis and Ki-67, a marker of tumor proliferation. Age was recorded in years, while Ki-67 was measured as a fraction, representing the proportion of cells exhibiting proliferation.

Post hoc power analysis was conducted to evaluate the study’s ability to detect significant effects. Given our sample size (n = 298), a significance level of 0.05, and effect size estimates from our logistic regression model, the power to detect associations between treatment protocol and recurrence was calculated to be 0.999. Similarly, the Pearson Chi-Square test had a power of 0.999, and the Mann–Whitney U test achieved a power of 0.990, assuming a moderate effect size (Cohen’s d = 0.5). These results confirm that the study was sufficiently powered to detect moderate-to-large effect sizes; however, some subgroup analyses, particularly those evaluating rare recurrence events within specific diagnostic groups, may have lower power due to smaller sample sizes.

### 3.1. Clinical and Pathological Characteristics Associated with Recurrence and Overall Survival

The analysis of categorical variables focused on recurrence and overall survival revealed critical insights into factors potentially influencing these outcomes. Diagnostic and treatment groups were thoughtfully categorized to identify clinically meaningful patterns. Diagnoses were grouped into categories such as local recurrence at study inclusion (RSI), axillary lymph node involvement, triple-negative breast cancer, progression, and primary tumors without metastasis. Chemotherapy protocols were stratified into anthracycline-based regimens, platinum/taxane-based regimens (including biologics), and other combinations. Regarding recurrence, the side of the affected breast did not exhibit a significant association (*p* = 0.87). Similarly, hormone receptor statuses (ER and PR) and HER2/neu positivity were not significantly different between patients with and without recurrence (*p* = 0.66, 0.98, and 0.46, respectively). These findings suggest that recurrence in this cohort may not be strongly influenced by these biomolecular characteristics. Tumor molecular subtypes showed no significant differences in distribution between groups (*p* = 0.51). While the Luminal B1 subtype appeared more frequent among patients with recurrence (37.21% vs. 24.71%), this difference was not statistically significant. This trend could indicate a potential link between this subtype and recurrence, which might warrant further investigation. Tumor grade (G classification) demonstrated a non-significant trend toward higher-grade tumors (G3) in the recurrence group (79.07% vs. 64.71%, *p* = 0.16). The predominance of high-grade tumors in the recurrence group aligns with the understanding that aggressive tumor biology is often associated with poorer outcomes, including higher recurrence rates and reduced survival. Diagnostic group comparisons revealed significant differences (*p* = 0.003). Axillary involvement in primary tumors was more frequent in the recurrence group (4.65% vs. 1.57%). Conversely, patients without recurrence predominantly had primary tumors without axillary involvement (52.16% vs. 27.91%). Additionally, RSI was significantly more common in the recurrence group (53.49% vs. 28.24%), whereas triple-negative disease showed a less pronounced difference (13.95% vs. 18.04%). These findings highlight the strong association between specific tumor characteristics and recurrence risk, emphasizing the need for targeted management strategies to improve patient outcomes. Treatment protocols revealed a borderline significant difference (*p* = 0.05). Anthracycline-based regimens were more common among patients with recurrence (97.67% vs. 87.84%), while platinum/taxane-based regimens (including biologics) were more prevalent in the no-recurrence group (12.16% vs. 2.33%). This suggests that the choice of chemotherapy may influence recurrence risk, though the direction of this effect and potential confounders require further study.

**Figure 1:**
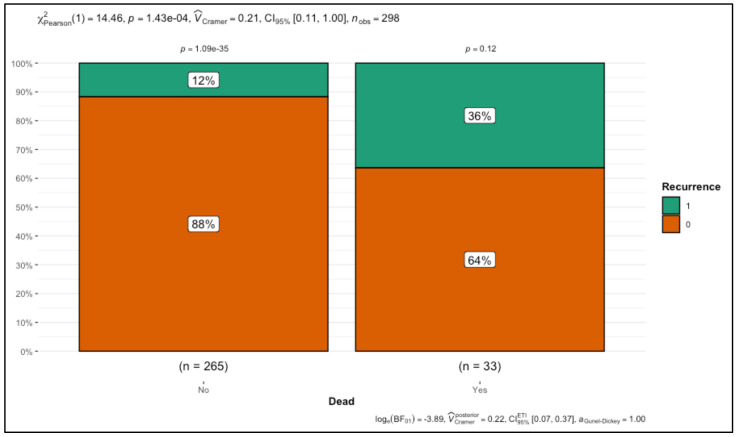
Association between recurrence status and mortality in breast cancer patients.

Overall survival was significantly poorer in the recurrence group, as evidenced by a higher mortality rate (27.91% vs. 8.24%, *p* < 0.001). This stark difference highlights the profound impact of recurrence on patient prognosis, reinforcing the importance of recurrence prevention in improving survival outcomes. The association between metastatic disease, treatment choice, and survival further illustrates the complexity of managing breast cancer patients at high risk for recurrence. The results suggest that recurrence is closely associated with metastatic disease and aggressive tumor features, while overall survival is significantly compromised in patients experiencing recurrence. Treatment protocols may influence recurrence and survival, necessitating further investigation into optimizing therapeutic strategies to reduce recurrence risk and enhance long-term outcomes. These findings provide critical insights for improving the management of breast cancer patients at high risk of recurrence and poor survival. The results are presented in Table 1 and Figure 1.

The analysis of continuous variables, including age at diagnosis and Ki-67, showed no significant differences between patients with and without recurrence. The median age at diagnosis was similar in both groups (54.00 years [IQR: 45.50–61.00] for no-recurrence vs. 53.00 years [IQR: 44.50–59.50] for recurrence, *p* = 0.43), indicating that age was not associated with recurrence risk in this cohort.

Similarly, the median Ki-67 value was 0.30 in both groups, with no significant difference in distribution (*p* = 0.67). This suggests that tumor proliferation, as measured by Ki-67, did not significantly predict recurrence in this study. The results are presented in Table 2.

### 3.2. Logistic Regression Analysis of Factors Associated with Recurrence

A logistic regression analysis was conducted to examine the association between clinical, pathological, and treatment-related variables and the odds of breast cancer recurrence. The model included key predictors such as hormone receptor status (ER and PR), HER2/neu positivity, Ki-67, treatment protocol, diagnostic group, and age at diagnosis, yielding a Nagelkerke R^2^ of 0.121, suggesting that the model explained approximately 12.1% of the variance in recurrence.

Estrogen receptor (ER) positivity was associated with increased odds of recurrence (OR = 1.52, 95% CI: 0.50–4.51, *p* = 0.447), though this result was not statistically significant. This aligns with prior findings indicating that ER-positive tumors can exhibit late recurrences, potentially due to their unique biological behavior and response to endocrine therapy. Conversely, progesterone receptor (PR) positivity was associated with a reduced recurrence risk (OR = 0.59, 95% CI: 0.22–1.65, *p* = 0.298). While not reaching statistical significance, this trend is consistent with literature suggesting PR positivity correlates with less aggressive tumor behavior and a more favorable prognosis.

HER2/neu positivity (OR = 0.71, 95% CI: 0.32–1.52, *p* = 0.387) and Ki-67 (OR = 0.76, 95% CI: 0.13–4.08, *p* = 0.754) did not demonstrate strong predictive effects in this model. While HER2-positive tumors are often associated with aggressive disease, targeted therapies such as trastuzumab likely mitigate recurrence risk. The limited effect of Ki-67 may be due to its variability within the cohort, reducing its predictive power.

The treatment protocol remained a key predictor in the multivariate model, adjusting for potential confounders such as hormone receptor status (ER, PR, HER-2/neu), Ki-67, diagnostic group, and age. The results indicate that platinum/taxane-based regimens (including biologics) were associated with a lower recurrence risk compared to anthracycline-based treatments (OR = 0.15, 95% CI: 0.01–0.78, *p* = 0.070), though this did not reach statistical significance. This suggests a potential protective effect of platinum/taxane-based chemotherapy, particularly in high-risk subgroups such as triple-negative disease or patients with residual disease after surgery.

While statistical significance was not reached, the trend observed supports the hypothesis that treatment choice may influence recurrence risk even after accounting for tumor biology and patient characteristics. Further studies with larger sample sizes may be necessary to confirm this relationship.

Regarding the diagnostic group, patients with primary tumors without axillary involvement showed a trend toward reduced recurrence risk (OR = 0.19, 95% CI: 0.03–1.47, *p* = 0.070), consistent with the expectation that localized disease has a more favorable prognosis. In contrast, RSI (OR = 0.70, 95% CI: 0.12–5.38, *p* = 0.694) and triple-negative disease (OR = 0.36, 95% CI: 0.05–3.36, *p* = 0.328) did not show statistically significant associations, likely due to the small sample sizes within these subgroups.

Age at diagnosis (OR = 0.98, 95% CI: 0.95–1.02, *p* = 0.331) did not significantly impact recurrence risk, suggesting that other tumor characteristics and treatment factors may be stronger predictors of recurrence than age alone.

Overall, the trends observed in this logistic regression analysis align with established clinical knowledge, highlighting the role of treatment protocols and disease presentation in recurrence risk. While several results did not reach statistical significance, this may be attributed to sample size limitations or subgroup variability. Notably, the strong association between platinum/taxane-based treatments and reduced recurrence risk warrants further investigation, as it could inform clinical decision-making to optimize outcomes in high-risk patients. The results are presented in Table 3.

**Figure 2:**
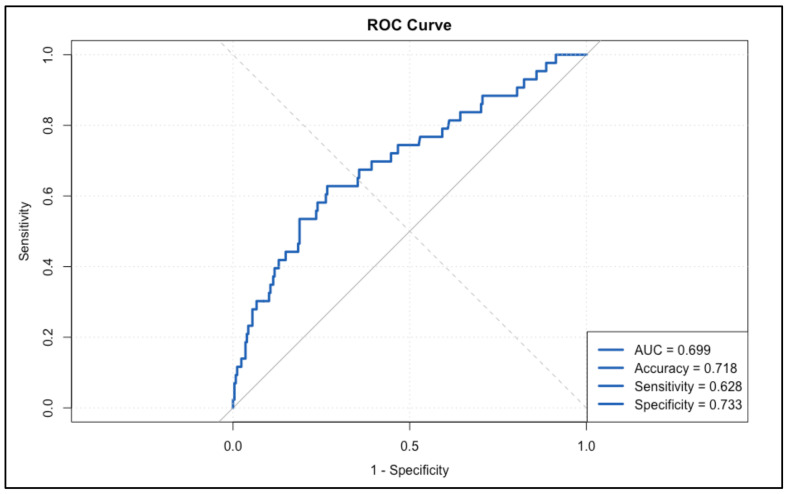
ROC curve for prediction of recurrence. Abbreviations: ROC—a receiver operating characteristic. AUC—area under the curve.

To evaluate the predictive performance of our model, we conducted a receiver operating characteristic (ROC) analysis. The area under the curve (AUC) was calculated as 0.6992, indicating acceptable discriminative ability in predicting recurrence. The ROC curve is shown in Figure 2.

### 3.3. Interaction Analysis of Predictors for Recurrence

To further explore the combined effects of key predictors on recurrence, interaction analyses were performed using logistic regression models. Interaction terms were introduced into the models to identify whether the relationship between certain predictors (e.g., treatment protocols, diagnostic groups, HER-2/neu status, and age) and recurrence risk varied depending on other variables. These analyses aimed to uncover potential synergistic or modifying effects that single-variable analyses may not capture. By examining interactions, we aimed to provide a more nuanced understanding of how clinical and pathological factors jointly influence recurrence outcomes in breast cancer patients.

**Figure 3:**
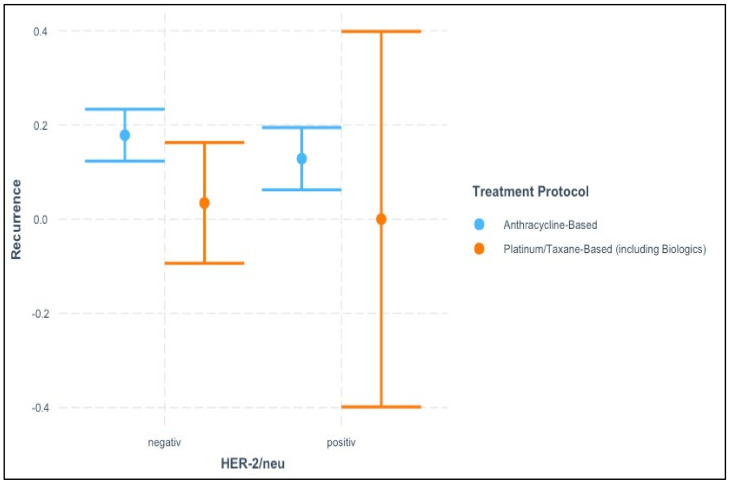
Interaction between HER-2/neu status and treatment protocol on recurrence risk.

The interaction analysis presented in Figure 3 examines the relationship between HER-2/neu status (negative vs. positive), treatment protocol (anthracycline-based vs. platinum/taxane-based, including biologics), and recurrence.

For HER-2/neu negative patients, recurrence rates were relatively low across both treatment protocols. However, a slight trend can be observed, with patients receiving platinum/taxane-based treatments showing slightly lower recurrence rates compared to those treated with anthracycline-based regimens. This suggests a potential benefit of platinum/taxane-based treatments in HER-2 negative patients, though the differences are subtle.

In contrast, for HER-2/neu-positive patients, there is a striking difference between treatment protocols. While anthracycline-based regimens maintained relatively consistent recurrence rates, the recurrence rate for patients treated with platinum/taxane-based protocols displayed substantial variability, as indicated by the wide confidence interval. This wide range could reflect either the small sample size of HER-2-positive patients receiving platinum/taxane-based treatments or a high level of heterogeneity in treatment responses within this group.

The interaction between treatment protocol and HER-2/neu status suggests that treatment efficacy may differ based on HER-2/neu status. Platinum/taxane-based regimens appear to have the potential to reduce recurrence in HER-2 negative patients, while the effect in HER-2 positive patients is less clear, possibly due to variability or small subgroup sizes. This interaction warrants further investigation in larger cohorts to clarify the role of HER-2/neu status in guiding treatment decisions and reducing recurrence risk.

The analysis of the relationship between age at diagnosis, progesterone receptor (PR) status, and recurrence reveals notable trends. Patients with PR-negative tumors exhibit a decline in recurrence rates with increasing age, suggesting that younger patients with PR-negative status are at greater risk for recurrence. This pattern may reflect the more aggressive tumor biology typically observed in younger patients with hormone receptor-negative breast cancer. Conversely, recurrence rates among PR-positive patients remain relatively stable across all age groups, indicating a potential protective effect of PR positivity. This stability suggests that PR-positive tumors may respond better to treatment and exhibit less aggressive behavior, contributing to more favorable outcomes regardless of patient age. These findings highlight the importance of considering both age and receptor status in recurrence risk assessment and treatment planning.

**Figure 4:**
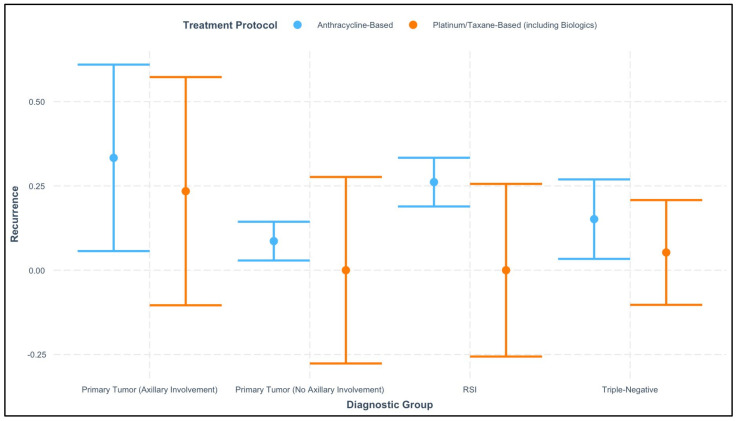
Interaction between diagnostic group and treatment protocol on recurrence rates.

Figure 4 presents an interaction analysis exploring the relationship between the diagnostic group, treatment protocol (anthracycline-based vs. platinum/taxane-based, including biologics), and recurrence. The plot illustrates variability in recurrence rates across diagnostic groups and treatment regimens, providing insights into potential interactions between disease characteristics and treatment efficacy.

For patients with axillary involvement, recurrence rates appeared elevated across both treatment protocols, with overlapping confidence intervals. This suggests that axillary involvement remains a strong predictor of recurrence, independent of the treatment regimen used. In contrast, for patients with primary tumors without axillary involvement, recurrence rates were markedly lower, and platinum/taxane-based regimens demonstrated a noticeable advantage over anthracycline-based treatments, suggesting a potential benefit of these regimens in early-stage disease.

Among patients classified as RSI, recurrence rates showed overlapping confidence intervals between the two treatment protocols, indicating no clear advantage of one regimen over the other in this group. For triple-negative breast cancer, platinum/taxane-based treatments were associated with slightly lower recurrence rates compared to anthracycline-based regimens. This observation aligns with existing evidence supporting the efficacy of platinum-based treatments in managing this aggressive subtype.

The findings for patients with progression revealed relatively low recurrence rates overall, but the wide confidence intervals in both treatment groups underscore the limited data available for this subgroup, making definitive conclusions challenging.

To evaluate whether HER2-targeted therapies (e.g., trastuzumab, pertuzumab) influenced the observed effects of platinum-based chemotherapy, we conducted an interaction analysis between HER2 status and chemotherapy protocol. In our interaction analysis, no statistically significant interaction was observed between HER2 status and platinum/taxane-based chemotherapy regarding recurrence risk (*p* = 0.9888). The effect estimate for the interaction term had a wide confidence interval, indicating high variability in treatment response within this subgroup. While platinum/taxane-based regimens were associated with lower recurrence risk in HER2-negative patients, the effect was less clear in HER2-positive patients, possibly due to the concurrent use of HER2-targeted therapies. These results suggest that further studies are needed to better delineate the independent contribution of platinum-based regimens in HER2-positive disease.

## 4. Discussion

This study provides valuable insights into the factors influencing breast cancer recurrence and overall survival in patients who underwent neoadjuvant chemotherapy and surgery, excluding those who had metastatic disease at initial diagnosis. By focusing solely on patients with non-metastatic disease at presentation, our findings offer a more refined analysis of recurrence risk and treatment efficacy within this population. While hormone receptor status and tumor molecular subtypes did not exhibit statistically significant associations with recurrence, trends observed in Luminal B1 and high-grade tumors suggest further research is needed to confirm their impact.

The molecular landscape of breast cancer is defined by specific biomarkers and signaling pathways that influence tumor growth, progression, and recurrence. This molecular complexity underpins the different clinical behaviors seen in breast cancer subtypes, and understanding these mechanisms is crucial for designing targeted therapies and improving patient outcomes. Breast cancer progression is governed by a variety of molecular pathways, including hormonal receptor signaling, growth factor receptor pathways, cell proliferation mechanisms, and defects in DNA repair systems. These pathways interact in complex ways, shaping the tumor’s behavior and response to treatment [7]. That is why currently we have a complex and well-coordinated interdisciplinary therapy that uses genomic markers such as Breast Cancer Gene 1/2 (BRCA1, BRCA2) and Phosphatidylinositol-4,5-Bisphosphate 3-Kinase Catalytic Subunit Alpha (PIK3CA), immunohistochemical markers like estrogen receptors (ER), progesterone receptors (PR) and human epidermal growth factor receptor 2 (HER2) and immunomarkers like Programmed Death-Ligand 1 (PD-L1) and tumor-infiltrating lymphocytes to determine the most suitable treatment for our patients and greatly improving their overall success rates [8,9,10,11]. Large rearrangements and deletions in the genes can also alter the function resulting simultaneously in an increased risk of breast and ovarian cancer [12]. There are different risk-reducing strategies and the most powerful strategy is risk-reducing surgery, represented by bilateral mastectomy and bilateral salpingo-oophorectomy [13,14,15].

Ki-67 is a nuclear protein associated with cellular proliferation. It is expressed during all active phases of the cell cycle but not in resting cells (G0 phase), making it a useful marker for assessing tumor growth rates. High Ki-67 expression is correlated with increased cell proliferation and is commonly found in aggressive breast cancer subtypes, such as luminal B, HER2-positive, and triple-negative (basal-like) cancers [16,17]. In particular, luminal B tumors exhibit higher Ki-67 levels compared to luminal A, reflecting a more proliferative and aggressive phenotype despite their hormone receptor positivity. The high proliferation rate indicated by Ki-67 leads to a shorter recurrence-free survival and worse prognosis [17]. Ki-67 is also used to guide treatment decisions, with high Ki-67 tumors being more likely to benefit from chemotherapy due to their rapid division rates [16,17].

The BRCA1 and BRCA2 genes are critical for DNA repair through the homologous recombination (HR) pathway, which repairs double-strand breaks. Mutations in BRCA1/2 lead to deficiencies in this repair mechanism, resulting in genomic instability and an accumulation of mutations that drive tumor development. BRCA1 mutations are more commonly associated with the basal-like (triple-negative) breast cancer subtype, which is known for its poor prognosis and high recurrence rates [7,8,18]. In BRCA-mutated cancers, the loss of DNA repair capacity makes these tumors highly sensitive to DNA-damaging agents such as platinum-based chemotherapies and PARP inhibitors, which exploit the tumor’s defective DNA repair machinery. However, some tumors eventually develop resistance to these therapies, often through the restoration of homologous recombination or alternative DNA repair pathways, leading to recurrence [8,18].

The St. Gallen International Breast Cancer Conference divided breast cancer into several molecular subtypes based on the expression of hormone receptors (ER, PR), HER2 status, and proliferation markers such as Ki-67 [19]. These subtypes are not only prognostic but also predictive of treatment responses. Luminal A: typically ER+/PR+, HER2−, and with low Ki-67 expression. These tumors grow more slowly, respond well to endocrine therapies, and have the lowest recurrence rates among all subtypes [16,19]. Luminal B: these tumors are ER+/PR+, but may be HER2-positive or -negative, with high Ki-67 expression indicating increased proliferative activity. Luminal B tumors are more aggressive than luminal A and have higher recurrence rates due to their faster growth and partial resistance to endocrine therapy. Luminal B1 breast cancer is a subtype within the broader Luminal B category, characterized by estrogen receptor (ER) positivity, variable progesterone receptor (PR) expression, HER2 negativity, and a high Ki-67 proliferation index. Whereas Luminal B2 is also HER2-positive [17,19]. HER2-Positive: Characterized by overexpression of HER2 and typically high proliferation rates, these tumors are aggressive but are often effectively treated with HER2-targeted therapies. Recurrence can occur due to resistance mechanisms involving downstream mutations in the PI3K/AKT/mTOR pathway [17]. Triple-Negative (Basal-like): lacking ER, PR, and HER2 expression, these tumors are highly aggressive and have poor prognoses. BRCA1/2 mutations are more frequently observed in this subtype, and while these tumors initially respond well to DNA-damaging agents, recurrence rates are high due to the development of resistance mechanisms [18,19,20].

Based on the study of biomarkers and having a classification of the different breast cancer intrinsic subtypes, the patients benefit from a better and more personalized therapy, and above all, the radical mastectomy is no longer necessary in all breast cancer patients, as long as complete removal of the tumor with free resection margins is performed, this being a prerequisite for a low risk of local recurrence [10].

Indications for breast-conserving treatment of breast cancer are: locally limited non-invasive breast cancer, invasive carcinomas with a favorable ratio of tumor size to breast volume, and invasive carcinomas with accompanying intraductal components, as long as the resection margins are healthy [21]. The standard breast cancer management nowadays includes neoadjuvant chemotherapy and conservative surgery, followed by adjuvant radiotherapy with or without adjuvant chemotherapy and/or endocrine therapy. Radiotherapy must be systematically performed, regardless of the characteristics of the disease, because it decreases the rate of local recurrence and consequently, specific mortality. A booster dose over the tumor bed is required if the patient is younger than 50 years old in order to reduce the local recurrence [22]. The aim of this study is to determine the importance of tumor biology and the impact of each molecular subtype on rates of breast cancer recurrence after neoadjuvant chemotherapy followed by breast-conserving surgery and radiotherapy. Several large-scale studies have demonstrated the efficacy of NAC in downstaging tumors and improving surgical outcomes. For instance, an extensive cohort study focused on predictors of locoregional recurrence (LRR) after NAC. The researchers developed nomograms incorporating factors such as age, clinical tumor size, nodal status before and after NAC, and pathologic response to predict the 10-year risk of LRR. Their findings emphasized that achieving a pathologic complete response (pCR) in both the breast and axillary nodes significantly reduces the risk of LRR, underscoring the importance of comprehensive pathologic assessment post-NAC [23]. Furthermore, a 20-year retrospective analysis identified predictive factors for overall survival (OS) and recurrence-free survival (RFS) in breast cancer patients receiving NAC. The study found that larger tumor size and nodal involvement adversely affected survival and recurrence rates, reinforcing the need for early detection and tailored treatment strategies [24].

Our results indicate that hormone receptor status (ER, PR) and HER2/neu expression did not demonstrate statistically significant associations with recurrence (*p* = 0.66, 0.98, and 0.46, respectively). These findings contrast with previous studies that have linked ER-positive tumors to a distinct recurrence pattern, particularly due to late recurrence risks associated with endocrine resistance [25,26]. However, recent advancements in targeted therapies, such as endocrine therapy and HER2-directed treatments, may have mitigated these effects, leading to reduced variability in recurrence outcomes. Nonetheless, our analysis suggests a possible trend towards increased recurrence in Luminal B1 tumors (37.21% in the recurrence group vs. 24.71%, *p* = 0.51), warranting further investigation into its clinical implications [27]. Tumor grade emerged as a potential risk factor, with a higher proportion of G3 tumors observed in the recurrence group (79.07% vs. 64.71%, *p* = 0.16). While not statistically significant, this trend aligns with previous research highlighting the aggressive nature of high-grade tumors and their association with increased proliferative activity, often reflected in higher Ki-67 expression levels [28]. In our cohort, Luminal B1 tumors demonstrated a higher recurrence trend (37.21% in the recurrence group vs. 24.71% in the non-recurrence group, *p* = 0.51), though this did not reach statistical significance. The potential clinical relevance of this finding is underscored by the role of Ki-67, a key marker of cellular proliferation. High Ki-67 expression has been correlated with poorer responses to endocrine therapy and increased recurrence risk in hormone receptor-positive breast cancer. While our study did not find a significant association between Ki-67 levels and recurrence (*p* = 0.67), the well-documented link between high Ki-67, endocrine resistance, and poor prognosis in Luminal B tumors suggests that further research is warranted to explore this connection in a larger cohort [29]. These findings reinforce the need for intensified treatment strategies for patients with high-grade tumors to mitigate recurrence risk [30,31,32]. Studies have shown that G3 tumors are associated with increased recurrence risk and poorer survival outcomes, particularly in hormone receptor-negative subtypes [33].

Our results also underscore the impact of chemotherapy regimens on recurrence rates. Patients treated with platinum/taxane-based regimens exhibited a lower recurrence risk compared to those receiving anthracycline-based regimens (*p* = 0.05). The role of platinum-based chemotherapy in HER2-positive patients remains complex due to the widespread use of HER2-targeted therapies, such as trastuzumab, which have significantly improved treatment outcomes. Our interaction analysis did not reveal a statistically significant modifying effect of HER2 status on the association between platinum/taxane-based chemotherapy and recurrence risk. The wide confidence interval for the interaction term suggests substantial variability, likely due to the small sample size in this subgroup. Given that HER2-targeted therapies are standard in HER2-positive breast cancer, it is challenging to determine whether the observed effects of platinum-based regimens in this group are independent of concurrent targeted therapy. Future studies with larger cohorts and detailed HER2-targeted therapy data are necessary to better assess the role of platinum-based chemotherapy in this setting. This finding is consistent with emerging evidence supporting the efficacy of platinum-based chemotherapy, particularly in triple-negative and other aggressive breast cancer subtypes [34]. However, alternative studies have suggested that anthracycline-free regimens may yield similar outcomes in select patient subgroups, emphasizing the need for personalized treatment approaches based on molecular profiling [35]. Further investigation into patient selection criteria for specific chemotherapy regimens is warranted to optimize clinical decision-making.

Prognostic outcomes in breast cancer are influenced by a multitude of factors beyond those analyzed in our study. One crucial aspect that strongly impacts prognosis is the pattern of residual disease following neoadjuvant therapy. The extent and molecular characteristics of residual disease serve as key determinants of recurrence risk and long-term survival. Recent literature highlights that patients with minimal residual disease exhibit significantly better outcomes compared to those with extensive residual tumor burden, particularly in aggressive subtypes such as triple-negative breast cancer (TNBC) and HER2-positive disease [36]. Moreover, the genomic and immune microenvironment of residual disease plays an essential role in therapy resistance and disease progression. Future studies should incorporate a detailed analysis of residual disease patterns, along with molecular profiling, to refine risk stratification and optimize post-neoadjuvant treatment strategies. Understanding these factors will aid in tailoring personalized therapeutic approaches to improve patient outcomes.

Our logistic regression analysis revealed a trend towards lower recurrence odds in PR-positive patients (OR = 0.59, *p* = 0.298). While not reaching statistical significance, this trend aligns with established evidence that PR expression is associated with a less aggressive tumor phenotype and improved responsiveness to endocrine therapy [37,38]. Our findings confirm that young patients with PR-negative disease exhibit an increased risk of recurrence, consistent with previous literature suggesting a more aggressive tumor biology in this subgroup. Given the known overlap between PR negativity and basal-like breast cancer, we explored this association within our cohort. While there was a trend toward a higher prevalence of basal-like subtype among young PR-negative patients, statistical significance was not reached, likely due to sample size limitations. Additionally, BRCA mutation status, a known contributor to aggressive PR-negative breast cancer, was not available in our dataset. Future studies incorporating BRCA genetic testing and a larger sample of young PR-negative patients will be essential to fully elucidate the molecular drivers of this observed recurrence risk [39].

Metastatic disease at initial diagnosis was strongly correlated with recurrence (*p* < 0.001). This result underscores the aggressive nature of tumors presenting with early metastasis, emphasizing the need for rigorous monitoring and tailored therapeutic strategies to prevent recurrence in high-risk groups.

Overall survival outcomes revealed a significant difference between recurrence and non-recurrence groups, with mortality rates of 27.91% and 8.24%, respectively (*p* < 0.001). These findings emphasize the substantial impact of recurrence on long-term prognosis and highlight the importance of aggressive recurrence prevention strategies.

Despite the valuable insights gained, this study has several limitations. The single-center, retrospective design may limit generalizability, and the sample size, particularly within certain subgroups, may have affected statistical power. Future research should focus on larger, multicenter prospective studies to validate these findings and further refine predictive models for recurrence risk.

One limitation of this study is the relatively small sample size in certain subgroups, particularly in the platinum/taxane-based chemotherapy group (n = 30) and the recurrence group (n = 43), which resulted in reduced statistical power for detecting smaller differences. While the observed trends provide valuable clinical insights, it is possible that some associations did not reach statistical significance due to limited sample size rather than a true absence of effect. A larger cohort would be necessary to confirm the statistical significance of these findings. Despite this limitation, the study provides an important exploratory analysis of chemotherapy protocols and recurrence risk in a real-world clinical setting, contributing to the understanding of treatment outcomes in breast cancer patients.

A key strength of our study is the inclusion of tumor grade and stage in our adjusted analyses, allowing for a more refined understanding of their impact on recurrence and survival outcomes. However, an important limitation is the lack of available data on surgical methods and adjuvant therapies such as radiotherapy and endocrine therapy, which could also influence patient outcomes. While these factors were not within the scope of our study, their absence should be considered when interpreting the results. Future studies incorporating these variables may provide further insights into optimizing treatment strategies.

Despite inherent limitations, this study contributes to the growing body of evidence on breast cancer recurrence by providing insights into non-metastatic cases. Future research should focus on integrating molecular biomarkers and genomic profiling into clinical decision-making to further personalize treatment strategies and improve long-term survival outcomes for breast cancer patients.

## 5. Conclusions

This single-center study highlights key predictors of breast cancer recurrence and overall survival in patients who underwent neoadjuvant chemotherapy followed by surgery, explicitly excluding those with metastatic disease at diagnosis. Our findings underline the crucial role of chemotherapy regimens and tumor biology in recurrence risk, along with patient age and hormonal factors, which significantly influence both recurrence and prognosis. Our results suggest that specific chemotherapy regimens may be associated with a reduced recurrence risk, particularly in aggressive breast cancer subtypes.

## Figures and Tables

**Table 1:** Comparison of categorical variables between patients with and without recurrence.

Variable	Group	No-Recurrence (n = 43)	Recurrence (n = 255)	*p*-Value
Affected breast	Left	139 (54.51%)	24 (55.81%)	0.87
	Right	116 (45.49%)	19 (44.19%)	
ER	Positive	145 (56.86%)	26 (60.47%)	0.66
PR	Positive	118 (46.27%)	20 (46.51%)	0.98
HER-2/neu	Positive	98 (38.43%)	14 (32.56%)	0.46
Luminal	Basal-like	73 (28.63%)	11 (25.58%)	0.51
	Luminal A	21 (8.24%)	2 (4.65%)	
	Luminal B1	63 (24.71%)	16 (37.21%)	
	Luminal B2	60 (23.53%)	9 (20.93%)	
	Non-Luminal	38 (14.9%)	5 (11.63%)	
G Classification	G1	4 (1.57%)	0 (0%)	0.16
	G2	86 (33.73%)	9 (20.93%)	
	G3	165 (64.71%)	34 (79.07%)	
Diagnostic Group	Primary Tumor (Axillary Involvement)	4 (1.57%)	2 (4.65%)	0.003
	Primary Tumor (No Axillary Involvement)	133 (52.16%)	12 (27.91%)	
	RSI	72 (28.24%)	23 (53.49%)	
	Triple-Negative	46 (18.04%)	6 (13.95%)	
Treatment Protocol	Anthracycline-Based	224 (87.84%)	42 (97.67%)	0.05
	Platinum/Taxane-Based (including Biologics)	31 (12.16%)	1 (2.33%)	
Died	Yes	21 (8.24%)	12 (27.91%)	<0.001

Abbreviations: ER—estrogen receptor. PR—progesterone receptor. HER-2/neu—human epidermal growth factor receptor 2. Luminal—breast cancer molecular subtypes. G Classification—tumor grade classification. Primary Tumor (Axillary Involvement)—presence of axillary lymph node metastases. Triple-Negative—triple-negative breast cancer subtype. RSI—recurrence at study inclusion. Treatment Protocol—chemotherapy protocol administered. Anthracycline-Based—treatment including anthracycline with or without taxane. Platinum/Taxane-Based (including Biologics)—chemotherapy including platinum agents, taxanes, and/or biologic agents. Died—mortality status; *p*-value—probability value for statistical hypothesis testing using the Pearson Chi-Squared test.

**Table 2:** Comparison of continuous variables between patients with and without recurrence.

Variable	No-Recurrence	Recurrence	*p*-Value
Age at diagnosis	54.00 (45.50–61.00)	53.00 (44.50–59.50)	0.43
Ki67	0.30 (0.20–0.50)	0.30 (0.20–0.45)	0.67

Abbreviations: Age at diagnosis—patient’s age at the time of breast cancer diagnosis in year; Ki67—Ki-67 proliferation index, reported as a fraction representing tumor cell proliferation; *p*-value—probability value for statistical hypothesis testing using the Mann–Whitney U test.

**Table 3:** Odds ratios for predictors of recurrence from logistic regression analysis.

Predictors	Odds Ratios	CI	*p*-Value
ER [Positive]	1.52	0.50–4.51	0.447
PR [Positive]	0.59	0.22–1.65	0.298
HER-2/neu [Positive]	0.71	0.32–1.52	0.387
Ki67	0.76	0.13–4.08	0.754
Treatment Protocol [Platinum/Taxane-Based (including Biologics)]	0.15	0.01–0.78	0.070
Diagnostic Group [Primary Tumor (No Axillary Involvement]	0.19	0.03–1.47	0.070
Diagnostic Group [RSI]	0.70	0.12–5.38	0.694
Diagnostic Group [Triple-Negative]	0.36	0.05–3.36	0.328
Age at diagnosis	0.98	0.95–1.02	0.331
R^2^ Nagelkerke = 0.121			

Abbreviations: ER—estrogen receptor. PR—progesterone receptor. HER-2/neu—human epidermal growth factor receptor 2. Ki67—Ki-67 proliferation index. reported as a fraction representing tumor cell proliferation. Treatment Protocol—chemotherapy protocol administered. Platinum/Taxane-Based (including Biologics)—chemotherapy including platinum agents, taxanes, and/or biologic agents. Diagnostic Group—classification based on disease presentation. Age at diagnosis—patient’s age at the time of diagnosis in years. CI—confidence interval. *p*-value—probability value for statistical hypothesis testing. R^2^ Nagelkerke—Nagelkerke’s R-squared value indicating model explanatory power.

## Data Availability

Further information concerning the present study is available from the corresponding author upon reasonable request.

## References

[1] World Cancer Research Fund International. https://www.wcrf.org/cancer-trends/breast-cancer-statistics/.

[2] Joshi A., GK A.V., Sakorikar T., Kamal A.M., Vaidya J.S., Pandya H.J. (2021). Recent advances in biosensing approaches for point-of-care breast cancer diagnostics: Challenges and future prospects. Nanoscale Adv..

[3] Bogdan R.G., Helgiu A., Cimpean A.M., Ichim C., Todor S.B., Iliescu-Glaja M., Bodea I.C., Crainiceanu Z.P. (2024). Assessing Fat Grafting in Breast Surgery: A Narrative Review of Evaluation Techniques. J. Clin. Med..

[4] Burciu O.M., Sas I., Popoiu T.A., Merce A.G., Moleriu L., Cobec I.M. (2024). Correlations of Imaging and Therapy in Breast Cancer Based on Molecular Patterns: An Important Issue in the Diagnosis of Breast Cancer. Int. J. Mol. Sci..

[5] Hübner J., Katalinic A., Waldmann A., Kraywinkel K. (2024). Long-term Incidence and Mortality Trends for Breast Cancer in Germany. Geburtshilfe Frauenheilkd..

[6] Subbiah S., Gopu G., Senthilkumar P., Muniasamy P. (2024). Molecular subtypes as a predictor of response to neoadjuvant chemotherapy in breast cancer patients. Indian J. Cancer.

[7] Curtis C., Shah S.P., Chin S.F., Turashvili G., Rueda O.M., Dunning M.J., Speed D., Lynch A.G., Samarajiwa S., Yuan Y. (2012). The genomic and transcriptomic architecture of 2000 breast tumours reveals novel subgroups. Nature.

[8] Ford D., Easton D.F., Peto J. (1995). Estimates of the gene frequency of BRCA1 and its contribution to breast and ovarian cancer incidence. Am. J. Hum. Genet..

[9] Cancer Genome Atlas Network (2012). Comprehensive molecular portraits of human breast tumours. Nature.

[10] Varzaru V.B., Anastasiu-Popov D.M., Eftenoiu A.E., Popescu R., Vlad D.C., Vlad C.S., Moatar A.E., Puscasiu D., Cobec I.M. (2024). Observational Study of Men and Women with Breast Cancer in Terms of Overall Survival. Cancers.

[11] Varzaru V.B., Vlad T., Popescu R., Vlad C.S., Moatar A.E., Cobec I.M. (2024). Triple-Negative Breast Cancer: Molecular Particularities Still a Challenge. Diagnostics.

[12] Shiovitz S., Korde L.A. (2015). Genetics of breast cancer: A topic in evolution. Ann. Oncol..

[13] Riis M.L. (2021). Management of patients with BRCA mutation from the point of view of a breast surgeon. Ann. Med. Surg..

[14] Cobec I.M., Popescu R., Moatar A.E., Verdes D. (2021). Ovarian cancer under the magnifying glass. Rom. J. Mil. Med. Vol..

[15] Cobec I.M., Sas I., Moatar A.E., Moleriu L., Rempen A. (2021). Ovarian cancer health politics in Romania and Germany: A comparative study. Exp. Ther. Med..

[16] Reddy P., Mithraa D.S. (2020). Correlation of ER, PR, Her2neu and Ki67 with other prognostic factors in breast carcinoma. Trop. J. Pathol. Microbiol..

[17] Loibl S., Poortmans P., Morrow M., Denkert C., Curigliano G. (2021). Breast cancer. Lancet.

[18] Turner N.C., Reis-Filho J.S. (2006). Basal-like breast cancer and the BRCA1 phenotype. Oncogene.

[19] Goldhirsch A., Wood W.C., Coates A.S., Gelber R.D., Thürlimann B., Senn H.J., Panel members (2011). Strategies for subtypes—Dealing with the diversity of breast cancer: Highlights of the St. Gallen International Expert Consensus on the Primary Therapy of Early Breast Cancer. Ann. Oncol. Off. J. Eur. Soc. Med. Oncol..

[20] Goldhirsch A., Winer E.P., Coates A.S., Gelber R.D., Piccart-Gebhart M., Thürlimann B., Senn H.J., Panel members (2013). Personalizing the treatment of women with early breast cancer: Highlights of the St Gallen International Expert Consensus on the Primary Therapy of Early Breast Cancer. Ann. Oncol. Off. J. Eur. Soc. Med. Oncol..

[21] Guidelinesprogramm Onkologie Interdisciplinary S3-Guidelines for the Early Diagnostic, Diagnostic, Therapy and Follow up of the Breast Cancer, Long Version 4.4, June 2021 AWMF-Registernummer: 032-045OL. http://www.leitlinienprogramm-onkologie.de/leitlinien/mammakarzinom/.

[22] Hennequin C., Belkacémi Y., Bourgier C., Cowen D., Cutuli B., Fourquet A., Hannoun-Lévi J.M., Pasquier D., Racadot S., Rivera S. (2021). Radiotherapy of breast cancer. Cancer Radiother..

[23] Mamounas E.P., Anderson S.J., Dignam J.J., Bear H.D., Julian T.B., Geyer C.E., Taghian A., Wickerham D.L., Wolmark N. (2012). Predictors of locoregional recurrence after neoadjuvant chemotherapy: Results from combined analysis of National Surgical Adjuvant Breast and Bowel Project B-18 and B-27. J. Clin. Oncol. Off. J. Am. Soc. Clin. Oncol..

[24] Chen D., Wang Q., Dong M., Chen F., Huang A., Chen C., Lu Y., Zhao W., Wang L. (2023). Analysis of neoadjuvant chemotherapy for breast cancer: A 20-year retrospective analysis of patients of a single institution. BMC Cancer.

[25] Zhu X., Ying X., Liu Y., Wang Y., Chen L., Shao Z., Jin X., Jiang Y., Wang Z. (2024). Stability and variability of molecular subtypes: Comparative analysis of primary and metastatic triple-negative breast cancer. Cancer Biol. Med..

[26] Liu Y., Teng L., Fu S., Wang G., Li Z., Ding C., Wang H., Bi L. (2021). Highly heterogeneous-related genes of triple-negative breast cancer: Potential diagnostic and prognostic biomarkers. BMC Cancer.

[27] Hanker A.B., Sudhan D.R., Arteaga C.L. (2020). Overcoming Endocrine Resistance in Breast Cancer. Cancer Cell.

[28] Ahn S.G., Bae S.J., Kim Y., Ji J.H., Chu C., Kim D., Lee J., Cha Y.J., Lee K.A., Jeong J. (2022). Author Correction: Primary endocrine resistance of ER+ breast cancer with ESR1 mutations interrogated by droplet digital PCR. NPJ Breast Cancer.

[29] Gluz O., Nitz U.A., Christgen M., Kuemmel S., Holtschmidt J., Schumacher J., Hartkopf A., Potenberg J., Lüedtke-Heckenkamp K., Just M. (2023). Efficacy of Endocrine Therapy Plus Trastuzumab and Pertuzumab vs De-escalated Chemotherapy in Patients with Hormone Receptor-Positive/ERBB2-Positive Early Breast Cancer: The Neoadjuvant WSG-TP-II Randomized Clinical Trial. JAMA Oncol..

[30] Ma Q., Liu Y.B., She T., Liu X.L. (2024). The Role of Ki-67 in HR+/HER2-Breast Cancer: A Real-World Study of 956 Patients. Breast Cancer Dove Med. Press.

[31] Inwald E.C., Klinkhammer-Schalke M., Hofstädter F., Zeman F., Koller M., Gerstenhauer M., Ortmann O. (2013). Ki-67 is a prognostic parameter in breast cancer patients: Results of a large population-based cohort of a cancer registry. Breast Cancer Res. Treat..

[32] Varzaru V.B., Eftenoiu A.E., Vlad D.C., Vlad C.S., Moatar A.E., Popescu R., Cobec I.M. (2024). The Influence of Tumor-Specific Markers in Breast Cancer on Other Blood Parameters. Life.

[33] Cancello G., Maisonneuve P., Rotmensz N., Viale G., Mastropasqua M.G., Pruneri G., Veronesi P., Torrisi R., Montagna E., Luini A. (2010). Prognosis and adjuvant treatment effects in selected breast cancer subtypes of very young women (<35 years) with operable breast cancer. Ann. Oncol. Off. J. Eur. Soc. Med. Oncol..

[34] Wang H., Zhang N., Sun Q., Zhao Z., Pang H., Huang X., Zhang R., Kang W., Shan M. (2024). Comparison of the efficacy of taxanes with carboplatin and anthracyclines with taxanes in neoadjuvant chemotherapy for stage II-III triple negative breast cancer: A retrospective analysis. J. Cancer Res. Clin. Oncol..

[35] Sharma P., Kimler B.F., O’Dea A., Nye L., Wang Y.Y., Yoder R., Staley J.M., Prochaska L., Wagner J., Amin A.L. (2021). Randomized Phase II Trial of Anthracycline-free and Anthracycline-containing Neoadjuvant Carboplatin Chemotherapy Regimens in Stage I-III Triple-negative Breast Cancer (NeoSTOP). Clin. Cancer Res. Off. J. Am. Assoc. Cancer Res..

[36] Tinterri C., Fernandes B., Zambelli A., Sagona A., Barbieri E., Di Maria Grimaldi S., Darwish S.S., Jacobs F., De Carlo C., Iuzzolino M. (2024). The Impact of Different Patterns of Residual Disease on Long-Term Oncological Outcomes in Breast Cancer Patients Treated with Neo-Adjuvant Chemotherapy. Cancers.

[37] Chaudhary L.N., Jawa Z., Hanif A., Szabo A., Kamaraju S., Cheng Y.C., Chitambar C.R. (2018). Does Progesterone Receptor Matter in the Risk of Recurrence for Patients With Ductal Carcinoma in Situ?. WMJ Off. Publ. State Med. Soc. Wis..

[38] Cancello G., Maisonneuve P., Rotmensz N., Viale G., Mastropasqua M.G., Pruneri G., Montagna E., Iorfida M., Mazza M., Balduzzi A. (2013). Progesterone receptor loss identifies Luminal B breast cancer subgroups at higher risk of relapse. Ann. Oncol. Off. J. Eur. Soc. Med. Oncol..

[39] Lee M.K., Varzi L.A., Chung D.U., Cao M.A., Gornbein J., Apple S.K., Chang H.R. (2015). The Effect of Young Age in Hormone Receptor Positive Breast Cancer. BioMed Res. Int..

